# Draft Genome Sequence of the First New Delhi Metallo-β-Lactamase (NDM-1)-Producing Escherichia coli Strain Isolated in Peru

**DOI:** 10.1128/genomeA.00199-18

**Published:** 2018-03-29

**Authors:** Jesus Tamariz, Carlos Llanos, Carlos Seas, Paola Montenegro, Jose Lagos, Miriam R. Fernandes, Louise Cerdeira, Nilton Lincopan

**Affiliations:** a Universidad Peruana Cayetano Heredia, Lima, Peru; b Clínica Oncosalud, Lima, Peru; c Clinica Delgado, Lima, Peru; d Departamento de Análises Clínicas, Faculdade de Ciências Farmacêuticas, Universidade de São Paulo, São Paulo, Brazil; e Department of Microbiology, Instituto de Ciências Biomédicas, Universidade de São Paulo, São Paulo, Brazil

## Abstract

We present here the draft genome sequence of the first New Delhi metallo-β-lactamase (NDM-1)-producing Escherichia coli strain, belonging to sequence type 155 (ST155), isolated in Peru. Assembly of this draft genome resulted in 5,061,184 bp, revealing a clinically significant resistome for β-lactams, aminoglycosides, tetracyclines, phenicols, sulfonamides, trimethoprim, and fluoroquinolones.

## GENOME ANNOUNCEMENT

Antibiotic resistance is a global problem with serious consequences for public health worldwide (1). In this regard, in Peru, high levels of antimicrobial resistance have been reported in recent years, particularly in Gram-negative bacteria, most belonging to the *Enterobacteriaceae* (2, 3). In the Latin American region, although the presence of New Delhi metallo-β-lactamase (NDM-1)-producing Gram-negative bacteria has been documented in Argentina, Brazil, Chile, Colombia, Ecuador, Paraguay, Peru, Uruguay, and Venezuela (4, 5), genomic studies conducted to characterize NDM producers have been limited (6–8). We hereby report the draft genome sequence of the first NDM-1-producing Escherichia coli strain (2ECMBL) in Peru, isolated in 2017 from a urine culture of an elderly patient with pancreatic cancer.

Genomic DNA of strain 2ECMBL was extracted using a PureLink quick gel extraction kit (Life Technologies, Carlsbad, CA) according to the manufacturer’s instructions. DNA quality and quantity were evaluated by agarose gel electrophoresis and by using a Qubit1 2.0 fluorometer (Life Technologies). The DNA library was prepared using a Nextera XT DNA library preparation kit (Illumina, Inc., Cambridge, UK), and genomic DNA was sequenced on an Illumina NextSeq PE platform using 150-bp paired-end reads, which yielded 345 contigs with an *N*_50_ value of 54,136 bp, a total number of assembled bases of 5,061,184 bp, and 84× coverage. *De novo* genome assembly was performed using Velvet v.1.2.10, and the contigs were annotated by the NCBI Prokaryotic Genome Annotation Pipeline (PGAP) v.3.2, whereas whole-genome sequence data were evaluated through bioinformatics tools (i.e., ResFinder v.3.0, VirulenceFinder v.1.5, MLST v.1.8, PlasmidFinder v.1.3, and pMLST v.1.4) available from the Center for Genomic Epidemiology (http://www.genomicepidemiology.org/).

Multilocus sequence typing (MLST) showed that carbapenem-resistant E. coli 2ECMBL belongs to sequence type 155 (ST155), whereas identification of plasmid replicons revealed the carriage of IncFIB and IncA/C2 plasmids, with the IncF sequence belonging to the K-:A-:B1- type. Using a 90% threshold, resistance genes to β-lactams (*bla*_PER-2_, *bla*_TEM-1B_, *bla*_NDM-1_, and *bla*_OXA-1_), phenicols (*catA2*, *cmIA1*, and *catB3*), rifampin (*arr-3*), sulfonamides (*sull*, *sul2*, and *sul3*), tetracyclines (*tetA*), trimethoprim (*dfrA31*), aminoglycosides [*aac(3)-Ia*, *aadA2*, *aadA1*, *aac(6´)lb-cr*, *strA*, *strB*, and *aac(3´)-lla*], and fluoroquinolones [*qnrVC1* and *aac(6´)Ib-cr*] were identified. In addition, the presence of the virulence genes *ipfA* (long polar fimbriae), *gad* (glutamate decarboxylase), and *cma* (colicin M) in the 2ECMBL strain was confirmed.

E. coli ST155 has been associated with humans and animals worldwide (9), and specifically in Latin America, this lineage has been shown to be shared between avian pathogenic *E. coli* (APEC) and human extraintestinal pathogenic *E. coli* (ExPEC) (10). Interestingly, to our knowledge, this is the first draft genome sequence of E. coli ST155 recovered from an infected human in this region, highlighting the cocarriage of multidrug resistance determinants, including the clinically significant β-lactamase resistance genes *bla*_NDM-1_ and *bla*_PER-2_.

In summary, this draft genome sequence provides valuable information that supports a better understanding and comparative genomic analysis of the dissemination of NDM-1-producing *Enterobacteriaceae* in clinical settings. In this regard, the emergence of strains of this sort in Peru is a cause for serious concern that supports the implementation of genomic surveillance studies in order to prevent the establishment of health care-associated infections (HAIs).

### Accession number(s).

The genome sequence of E. coli strain 2ECMBL has been deposited in DDBJ/ENA/GenBank under the project accession number PISG00000000.
